# Towards best use and regulatory acceptance of generic physiologically based kinetic (PBK) models for in vitro-to-in vivo extrapolation (IVIVE) in chemical risk assessment

**DOI:** 10.1007/s00204-022-03356-5

**Published:** 2022-09-05

**Authors:** Abdulkarim Najjar, Ans Punt, John Wambaugh, Alicia Paini, Corie Ellison, Styliani Fragki, Enrica Bianchi, Fagen Zhang, Joost Westerhout, Dennis Mueller, Hequn Li, Quan Shi, Timothy W. Gant, Phil Botham, Rémi Bars, Aldert Piersma, Ben van Ravenzwaay, Nynke I. Kramer

**Affiliations:** 1 Beiersdorf AG, Hamburg, Germany; 2 Wageningen Food Safety Research, Wageningen, The Netherlands; 3 Center for Computational Toxicology and Exposure, Office of Research and Development, U.S. Environmental Protection Agency, Research Triangle Park, NC USA; 4 esqLABS GmbH, Saterland, Germany; 5 The Procter and Gamble Company, Cincinnati, OH USA; 6 National Institute for Public Health and the Environment, Bilthoven, The Netherlands; 7 Corteva Agriscience, Indianapolis, IN USA; 8 The Dow Chemical Company, Midland, MI USA; 9 The Netherlands Organisation for Applied Scientific Research TNO, Utrecht, The Netherlands; 10 Research and Development, Crop Science, Bayer AG, Monheim, Germany; 11 Unilever Safety and Environmental Assurance Centre, Colworth Science Park, Sharnbrook, Bedfordshire UK; 12 Shell Global Solutions International B.V, The Hague, The Netherlands; 13 School of Public Health, Faculty of Medicine, Imperial College London, London, UK; 14 Syngenta, Jealott’s Hill, Bracknell, Berkshire UK; 15 Crop Science Division, Bayer S.A.S., Sophia Antipolis, France; 16 Environmental Sciences Consulting, Altrip, Germany; 17 Toxicology Division, Wageningen University, PO Box 8000, 6700 EA Wageningen, The Netherlands

**Keywords:** IVIVE, PBK models, NAMs, Regulatory acceptance, Risk assessment

## Abstract

With an increasing need to incorporate new approach methodologies (NAMs) in chemical risk assessment and the concomitant need to phase out animal testing, the interpretation of in vitro assay readouts for quantitative hazard characterisation becomes more important. Physiologically based kinetic (PBK) models, which simulate the fate of chemicals in tissues of the body, play an essential role in extrapolating in vitro effect concentrations to in vivo bioequivalent exposures. As PBK-based testing approaches evolve, it will become essential to standardise PBK modelling approaches towards a consensus approach that can be used in quantitative in vitro-to-in vivo extrapolation (QIVIVE) studies for regulatory chemical risk assessment based on in vitro assays. Based on results of an ECETOC expert workshop, steps are recommended that can improve regulatory adoption: (1) define context and implementation, taking into consideration model complexity for building fit-for-purpose PBK models, (2) harmonise physiological input parameters and their distribution and define criteria for quality chemical-specific parameters, especially in the absence of in vivo data, (3) apply Good Modelling Practices (GMP) to achieve transparency and design a stepwise approach for PBK model development for risk assessors, (4) evaluate model predictions using alternatives to in vivo PK data including read-across approaches, (5) use case studies to facilitate discussions between modellers and regulators of chemical risk assessment. Proof-of-concepts of generic PBK modelling approaches are published in the scientific literature at an increasing rate. Working on the previously proposed steps is, therefore, needed to gain confidence in PBK modelling approaches for regulatory use.

## Introduction

In vitro tests using human organotypic cells and tissue are at the basis of next generation risk assessment (Bell et al. 2018; Bessems et al. 2014; Breen et al. 2021; Coecke et al. 2013; Krewski et al. 2020; NRC 2007; Rotroff et al. 2010; Wetmore 2015). The application of these in vitro assays in quantitative hazard characterisation depends on the accurate extrapolation of concentration–response relationships from in vitro to in vivo*,* a process referred to as in vitro to in vitro extrapolation (IVIVE) (Kulkarni et al. 2021). The process entails translating in vitro concentration–response relationships to an external dose–response relationship in humans and animals, like an oral dose in a repeat dose pharmacological or toxicological study. There are different kinds of IVIVE. IVIVE may refer to extrapolating kinetic parameters measured in vitro to estimates of the associated kinetics in vivo: for example, an intrinsic clearance rate measured in vitro in human liver microsomes needs to be extrapolated to estimate in vivo whole-liver human hepatic clearance rate. Here, however, we specifically refer to quantitative IVIVE (QIVIVE) as the process of translating in vitro concentration–response relationships to in vivo dose–response relationships in humans and animals (Blaauboer 2010; Louisse et al. 2017; Yoon et al. 2012). Physiologically based kinetic (PBK) models describing the absorption, distribution, metabolism and excretion (ADME) of the chemical in the organism provide a means for QIVIVE (Jones and Rowland-Yeo 2013; Kuepfer et al. 2016; Pearce et al. 2017b; Punt et al. 2021b; Wetmore 2015). PBK models consist of selected interconnected tissue compartments through which a chemical of interest is processed. In its simple form, the PBK model predicts concentrations of the chemical in each tissue compartment as results from the (1) blood flow rates, (2) concentration of the chemical in the incoming blood (or plasma), (3) volume of tissue, (4) equilibrium tissue to blood (or tissue to plasma) partition coefficients, and (5) any clearance (removal or transformation) of the chemical from the tissue. When applying the PBK model in a “reverse dosimetry” fashion, the PBK models are used to infer external equivalent exposure regimes that would cause blood, plasma, or target tissue concentrations equal to bioactive concentrations identified by in vitro assays (Louisse et al. 2017; Rotroff et al. 2010; Tan et al. 2007; Yoon et al. 2012).

Historically, PBK models were developed for specific chemicals and for purposes other than QIVIVE. A so-called top-down approach was used by designing the model structure and fitting its parameters to reproduce specific in vivo experimental animal or human kinetic studies (Bessems et al. 2014). These PBK models were designed to allow extrapolation from data collected in certain situations by estimating chemical-specific parameters and then using knowledge of physiology to make predictions for other scenarios without kinetic data (for example in different species or exposures by other routes) (Andersen 1995; Chiu et al. 2007). The selection of which aspects of physiology to include in these PBK models was often a function of both the extrapolation needed as well as expert determination of the processes key to understanding the specific chemicals kinetics, for example transporters for chemicals known to be substrates or lungs for chemicals known to be volatile (Andersen 1995; Campbell et al. 2012; Clewell et al. 2000; Jones et al. 2012a). Within the last decade, however, an important shift has taken place towards so-called bottom-up approaches that make use of generic model structures (that is, describing the same aspects of physiology for all chemicals) and in vitro and/or in silico input data to parameterise these models (Bessems et al. 2014; Breen et al. 2021; Cohen Hubal et al. 2019; Honda et al. 2019; Jamei et al. 2009; Pearce et al. 2017b; Punt et al. 2021a; Rodgers and Rowland 2006; Wambaugh et al. 2018). There were several important drivers for this shift towards bottom-up approaches. Within the pharmaceutical domain there was a need to reduce the number of failures in drug development by selecting favourable kinetic characteristics in an earlier stage (Edginton et al. 2008; Jones et al. 2012b; Stillhart et al. 2019; Wang 2010). For non-therapeutic chemicals, there was need for risk assessment strategies that are less reliant on in vivo toxicokinetic data for both humans and model animals (Bell et al. 2018; Breen et al. 2021; Rotroff et al. 2010; Sipes et al. 2017; Tonnelier et al. 2012; Wambaugh et al. 2015; Wetmore et al. 2012). Additionally, it was gradually recognised that consistent model implementations are more reliable and verifiable (Breen et al. 2021; Clark et al. 2004; Cohen Hubal et al. 2019; Eissing et al. 2011; Jamei et al. 2009; McLanahan et al. 2012; Ruiz et al. 2011; Tan et al. 2020). Technical advancement, particularly the increasing availability of chemical-specific in vitro kinetic data and the in vitro to in vivo scalars for interpreting these data, have also led to this shift towards bottom-up approaches for PBK model development (Choi et al. 2019; Jamei 2016).

The shift towards bottom-up modelling approaches has resulted in substantial reduction in model structure from one chemical to another. The bottom-up PBK models can be chemical-specific or generic. The development of a chemical-specific bottom-up PBK model requires understanding of the physiochemical and ADME properties and target organ of interest (Hoffman and Hanneman 2017; Najjar et al. 2021; Pletz et al. 2020). Each of these relevant processes are simulated based on in vitro and in silico input data. On the other hand, generic PBK models generally apply only limited number of input parameters, particularly liver metabolism and distribution parameters like tissue:partition coefficients, to obtain first estimates of the kinetics of multiple chemicals (Jongeneelen and Berge 2011; Peters 2008; Zhang et al. 2019). Different platforms are available that provide a user-friendly interface around bottom-up PBK model approaches, including commercial and open-source/freeware, such as GastroPlus®, Simcyp™, PK-Sim®, MEGEN/RVIS, and IndusChemFate. Bottom-up PBK tools are also available as function libraries for computer programming languages for example, httk, PLETHEM, and SimBiology (Breen et al. 2021; Hack et al. 2020; Pearce et al. 2017b).

The societal request to reduce animal testing, and therefore the increased reliance on in vitro data for quantitative risk assessment demonstrate the necessity for good and reliable QIVIVE models. As indicated above, the PBK model stands at the very centre of QIVIVE modelling, taking into account the number of chemicals which will need to be assessed by New Approach Methodologies (NAMs) if animal testing is reduced significantly. PBK model development should become as efficient as possible. Therefore, it is essential that generic PBK models based on limited input data are available for such purposes. However, such models, efficient as they may be, will also need to be reliable and their applicability domain will need to be defined (Honda et al. 2019; Parish et al. 2020; Wambaugh et al. 2015; Wetmore et al. 2013). Given the broad chemical space that needs to be covered, a single generic PBK model is unlikely to fulfil regulatory requirements for a reliable QIVIVE for all chemicals. Rather, defining a group of complementary PBK models with defined chemical applicability domains seems a more realistic approach towards a consensus strategy for QIVIVE in the regulatory context. Advancement towards such a consensus strategy needs definition of regulatory requirements, and PBK models meeting those requirements. Given the availability of several PBK models, software and approaches, a general strategy is required to standardising generic PBK models and for defining their application in chemical hazard and risk assessment. To work towards the general strategy to facilitate the regulatory acceptance of using PBK models for chemical risk assessment, The European Centre for Ecotoxicology and Toxicology of Chemicals (ECETOC) invited experts from different sectors to address this strategy, in a 2-day workshop (9–10 November 2021). This article summarises the outcome of the discussion based on the following questions:

I. What are the commonalities and differences in these models?
II. What are the critical input parameters to existing and new models?
III. How can we make existing model domains reliable and acceptable to regulators?

### Commonalities and differences in structure of bottom-up PBK models for QIVIVE

Over the last decade, an increasing number of platforms have become available to perform PBK model simulations. A summary of the different tools has been reported by Hack et al. (2020). A survey amongst the PBK modelling community showed that amongst the different modelling platforms, Berkeley Madonna, R, and MATLAB are currently most frequently used for model building (Hack et al. 2020; Paini et al. 2017). These are general-purpose differential equation solvers that require the definition of the model equations by the user. GastroPlus®, SimCyp™, PK-Sim® are the frequently used platforms that allow model building with a graphical user interface (Hack et al. 2020; Paini et al. 2017).

These platforms differ in terms of flexibility in the model structure, whether the platform is open source or not, the number of species or exposure routes that can be simulated, the extent of parameterisation required, and whether it contains a graphical user interface or requires programming skills (Hack et al. 2020). In addition, differences between platforms occur with respect to the available physiological data and whether human variability or special populations can be simulated. The number of compartments that are included in a model can also be different, ranging from 6-compartment models (for example httk) to models that contain all major organs of the body (for example SimCyp, GastroPlus, PK-Sim) with different granularity of details per organ. For all platforms, the underlying models, software implementation, and parameterizing data used can in principle be identified; however, for commercial platforms, this information is only shared under confidentiality conditions with regulatory bodies.

Despite the differences between platforms, the concept of the bottom-up modelling approaches will be very similar. Regardless of platform, the model will generally include compartments describing the key organs involved in ADME, and descriptions of key ADME processes: intestinal absorption (for oral exposure), liver metabolism, partitioning in different tissues, and the fraction unbound are usually included as the first input parameters to make initial estimates of internal exposure of a chemical (Jones and Rowland-Yeo 2013). When needed, additional kinetic processes like extrahepatic metabolism or transporter-mediated processes are included, which are important for some xenobiotics (Cooper et al. 2019). It depends on the platform, in which additional inputs need to be included. In addition, with general purpose differential equation solvers, each additional kinetic process needs to be manually added to the model code.

**Fig. 1:**
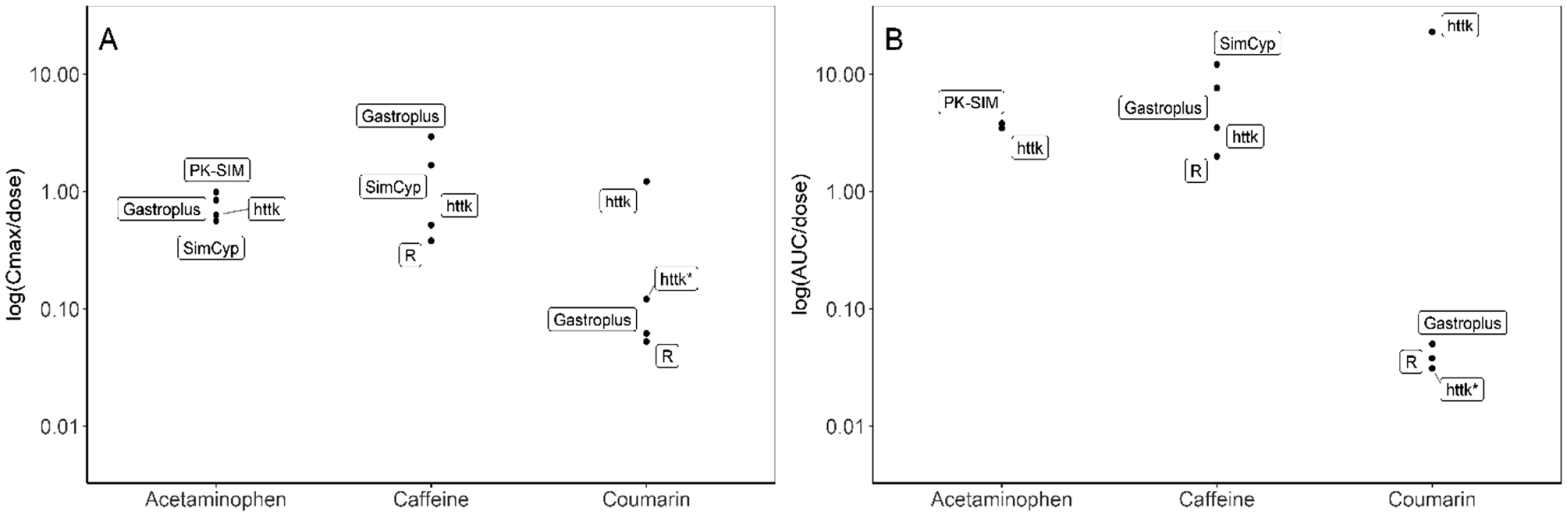
Cmax (**A**) and AUC (**B**) predictions reported in the literature as obtained with different PBK modelling platforms after a single oral dose. In case of httk, the Cmax and AUC predictions were generated with the httk (v2.0.0) package in R (4.1.1) (Pearce et al. 2017b). The remaining PBK model results were obtained from different scientific publications. In case of acetaminophen, the data were obtained from (Gaohua et al. 2016; Mian et al. 2020; Pearce et al. 2017b; Statelova et al. 2020). For caffeine, the PBK-predicted plasma levels were obtained from (Gajewska et al. 2014; Gaohua et al. 2016; Mian et al. 2020; Pearce et al. 2017b) and for coumarin from (Gajewska et al. 2014; Moxon et al. 2020; Pearce et al. 2017b). Both the Cmax and AUC values were normalised to the dose to be able to compare the results from the different publications. In case of coumarin, the httk Cmax and AUC predictions were performed with an in vitro measured liver intrinsic clearance of 0 L/h as well as an in vitro measured intrinsic clearance value of 929 L/h as obtained from (Moxon et al. 2020). The latter simulations are marked with an asterisk

Given that the physiology and differential equations of bottom-up PBK modelling approaches within different platforms are similar, differences in results are generally not due to the platform that is used, but due to the choices that users make and the input data that are included. Recently, Ahmad et al. (2020) compared half-lives (*t*_1/2_), maximum plasma concentrations (Cmax) and area under the plasma concentration–time curve (AUC) obtained with three different software packages (GiSim, GastroPlus and Simcyp) for a range of pharmaceutical compounds. They observed that the predicted AUC_0-tlast_, Cmax, and *t*_1/2_ of 62–75% of the 58 selected compounds were within threefold range of the observed values in humans. In addition, they observed that the average predictive performance did not relate to the software package that was used (Ahmad et al. 2020). Potential differences in model predictions between different PBK modelling platforms can also be seen in Fig. 1, which shows literature reported Cmax and AUC predictions (normalised to the dose) for acetaminophen, caffeine, and coumarin, that are each obtained with different platforms. The simulations are generally within a narrow range. Although the Cmax and AUC predictions are overall close to each other, the results of Fig. 1 also reveal that, in case of coumarin, the Cmax and AUC predictions are substantially different when using the results from the httk package (Pearce et al. 2017b) compared with the other platforms. The httk package uses an intrinsic liver metabolic clearance estimate of 0 L/h for coumarin, based on in vitro measurements with human hepatocytes (Wetmore et al. 2015). The other publications use estimates of 929 L/h, scaled from in vitro measurements with human hepatocytes (Moxon et al. 2020), and 634 L/h, scaled from in vitro human liver (Gajewska et al. 2014; Rietjens et al. 2008). When the httk model is run using the estimate from Moxon et al. (2020), its Cmax and AUC predictions agree with the other modelling platforms. This shows the impact that the choice of input parameter values has on model predictions, and therefore the quality of in vitro and in silico studies used to derive these values. This result also highlights the need for probabilistic approaches that account for variability and uncertainty in data (in vitro*, *in vivo, model platform, etc.) to provide a range of predictions.

### Important input parameters

Input parameters in PBK models for QIVIVE can be categorised into three main groups: (1) chemical-specific parameters, (2) anatomical and physiological parameters such as organ volumes and blood flow rates, and (3) exposure properties such as in vitro response concentrations and exposure regimen (Kuepfer et al. 2016; Madden et al. 2019; Shebley et al. 2018). When considering generic PBK modelling approaches, the anatomical and physiological parameters are part of the model code or platform. The values generally represent the average of a population or, depending on the platform, also include the human variability or representative values for special populations like children. In addition, generic PBK models for various animal species (e.g. mouse, rat, dog) are available and contain the anatomical and physiological data of the animal.

An overview of important chemical-specific parameters and approaches to obtain these parameters is provided in Table 1. Parametrisation of bottom-up PBK models for a specific chemical generally starts with the collection of physicochemical properties, including the log P and p*K*a. These are used as input for calculating partition coefficients, for which different approaches exist (Table 1). The intrinsic liver clearance is also one of the first parameters that is included in a PBK model (Jones and Rowland-Yeo 2013), which can for example be measured in incubations with (cryopreserved) primary hepatocytes or liver microsomal or S9 fractions. Depending on the route of exposure, the dermal, inhalation or oral absorption rates need to be included in the model. Choi et al. indicate that oral absorption is well predicted using in vitro Caco-2 or PAMPA absorption studies, given for chemical within the applicability domain of the models, both biologically (e.g. expression of necessary transporters) and chemically (e.g. lipophilicity range) (Choi et al. 2019). Dermal absorption may be characterised using the In Vitro Permeation Test (IVPT) that uses ex-vivo human skin or pig skin (Santos et al. 2020). If needed, additional kinetic processes, such as transporter-mediated influx or efflux in the intestine, liver and kidney are included in the model structure. For most in vitro-derived input parameters, scaling factors are required, corresponding to the relative expression or activity factor [REF, RAF (Choi et al. 2019)], the ratio of the number of cells per tissue weight (for example, the PTCPGK, proximal tubule cells per gramme kidney) and tissue mass. In case of transporter-mediated processes, this scaling of in vitro kinetic data is still challenging. For example, the preservation of expression and activity levels in in vitro renal cell models is poor, variable and often unknown, hampering their use for QIVIVE (Scotcher et al. 2016).

**Table 1:** Chemical-specific input parameters for PBK models obtained by in vitro methods or calculated using in silico approaches

Parameter	Frequently applied approach	Example protocols or software
Liver intrinsic clearance	In vitro: S9/liver microsomes or hepatocytes	Richardson et al. (2016); Shibata et al. (2000)
	In silico	ADMET Predictor (SimulationsPlus), Dawson et al. (2021); Pradeep et al. (2020)
Extrahepatic metabolism (e.g. intestine, kidney, lungs)	In vitro: S9 or microsomes	Cubitt et al. (2011); Richardson et al. (2016)
Partition coefficients, for example tissue:plasma	In silico	Berezhkovskiy (2004); Pearce et al. (2017a); Rodgers and Rowland (2006); Schmitt (2008); SimulationsPlus
Fraction unbound in plasma (fu)	In vitro: equilibrium dialysis	Ryu et al. (2021)
	In silico	Dawson et al. (2021); Lobell and Sivarajah (2003); Pradeep et al. (2020); SimulationsPlus; Yun et al. (2021); Zhu et al. (2013)
Intestinal uptake and efflux	Caco-2 or PAMPA permeability studies	Choi et al. (2019)
	In silico	Hou et al. (2004)
Dermal uptake	In Vitro Permeation Test (IVPT) that uses ex-vivo human skin or pig skin	Santos et al. (2020)
	In silico	Dancik et al. (2013); Guy and Potts (1993)
Transporter kinetics	Transporter-transfected cell lines with single transporters or multiple transporters	Wang (2019)
	In silico	Sedykh et al. (2013); SimulationsPlus

It should be noted that not all parameters depicted in Table 1 need to be included for every combination of chemical and exposure scenario being modelled. The most critical parameters for a specific chemical depend on (1) the site of exposure (e.g. oral, dermal, inhalation), (2) the kinetic processes that are hypothesised to be needed to accurately simulate the ADME profile of a chemical (e.g. if a chemical is cleared predominantly through active secretion in the proximal tubule, elimination in the kidney is not accurately simulated using solely the Glomerular Filtration Rate, GFR), and (3) sensitive parameters included in the PBK model that, when changed, have the greatest influence on the model output (e.g. human bioequivalent exposure levels). To determine the impact of specific parameters on model predictions, sensitivity analyses are essential as these allow the variation of model outputs to be ascribed to the uncertainty (or other variability) of the inputs to the model.

Experience working with simple generic PBK models, such as the IndusChemFate model, httk and www.qivivetools.wur.nl, as well as more complex proprietary models, such as GastroPlus® and Simcyp™ PBPK Simulator, provides an insight into critical parameters and how best to obtain these. Over the past decade chemical-specific measurements for key parameters have been made publicly available for both the human and the rat for more than a thousand chemicals (Honda et al. 2019; Rotroff et al. 2010; Tonnelier et al. 2012; Wambaugh et al. 2019; Wetmore et al. 2015, 2013, 2012). These form a basis for a strategy for PBK model optimisation and selection for QIVIVE applications that covers the wide spectrum of chemical entities and is applicable to in vitro assays that represent all elements of chemical safety testing.

Aside from kinetic input parameters to develop PBK models, the type of in vitro toxicity data as input to calculate external exposure levels that are safe/toxic to humans is critical for adequate risk assessments. In vitro toxicity readouts are biomarkers for molecular perturbations in toxicity pathways. Organism-level Adverse Outcome Pathway (AOP) models are required to extrapolate in vitro toxicity pathway perturbation to in vivo Points of Departure (POD). Organism-level Adverse Outcome Pathway (AOP) models are required to extrapolate in vitro toxicity pathway perturbation to in vivo Points of Departure (POD). Defining relevant AOPs for a particular chemical and relating a magnitude of a molecular perturbation to a magnitude of alteration in an apical endpoint is a major challenge and requires, amongst others, batteries of in vitro assays that screen the entire toxicity pathway space (Zhang et al. 2018).

Moreover, it is critical to determine the appropriate dose metrics both in vitro and in vivo. In in vitro*,* nominal effect concentrations measured after 24 h exposure are generally used in QIVIVE studies, despite (1) chemicals differentially binding to in vitro system components, such as serum constituents and plastic, which reduces the concentration at the molecular target in vitro, and (2) many transcriptionally mediated cellular stress responses peaking at various times depending on the genes, chemical and chemical concentration (Groothuis et al. 2015; Zhang et al. 2018). In vivo*,* plasma or tissue Cmax (maximal concentration) are commonly used dose metrics to relate systemic exposure to toxicological effects in vivo. Whether this is appropriate is dependent on the chemical’s mechanism of action (Groothuis et al. 2015; McNally et al. 2018; Punt et al. 2021b). For this reason, Groothuis et al. and Li et al. argue that the decision to use Cmax, AUC or other dose metrics in vivo and in vitro should be determined on a case-by-case basis, for which guidelines need to be developed (Groothuis et al. 2015; Li et al. 2021).

### Acceptance by regulators

**Fig. 2:**
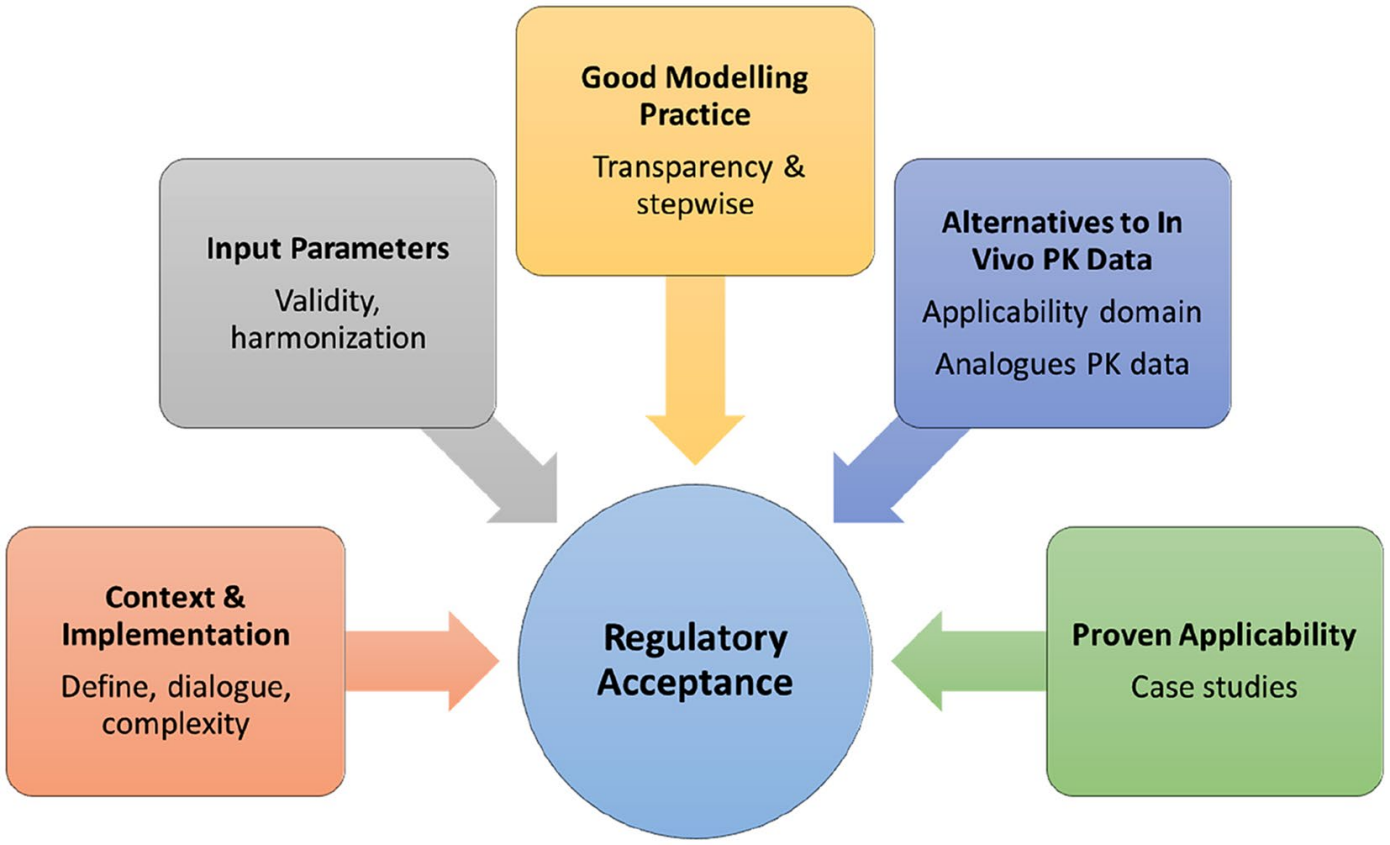
Considerations influencing regulatory acceptance of PBK models

Although increasingly more proof-of-concepts of bottom-up PBK modelling approaches are available to the scientific community (Basketter et al. 2012; Coecke et al. 2013; Gajewska et al. 2014; OECD 2020a, b, 2021), challenges remain regarding the acceptance of PBK model results in regulatory risk assessments (Bopp et al. 2019; Paini et al. 2021a; Wambaugh et al. 2019). For example, the use of generic PBK models to prioritise chemicals with the highest likelihood of causing health effects is accepted by regulators to varying degrees and depends on the risk assessment application and context (Chebekoue and Krishnan 2019). Comparisons with in vivo animal or human reference data are generally required to demonstrate fit-for-purpose adequacy. However, for many chemicals, particularly outside the pharmaceutical domain, such reference data are difficult to obtain. To overcome the lack of data, several alternative steps are recommended that can contribute to improving regulator adoption. These are presented in Fig. 2 and include (1) the definition of context and implementation because model complexity is driven by the required level of confidence, (2) the harmonisation and validity of input parameters, (3) the establishment of good modelling practice (GMP), (4) proof of the predictive power using alternatives to in vivo PK data, for example a read-across approach, and (5) conducting case studies to prove the applicability of the PBK models outcomes.

*Defining context, step 1* In human health risk assessment, any lack of data may be addressed if plausible conservative assumptions can be identified. These assumptions might provide a degree of conservatism sufficient to allow for human health protection. However, such assumptions may not always be available or may vary depending on context (for example, assuming 100% oral absorption is conservative for reverse dosimetry but underestimates potency when predicting tissue concentrations in toxicological studies) (Bell et al. 2018). Increasing model complexity is unlikely to improve predictive performance and regulatory acceptance. Instead, fewer mechanisms and fewer parameters often lead to more accurately characterised uncertainties (Chiu et al. 2007; Jamei 2016; Rowland et al. 2004). The principle of parsimony becomes essential—generic models should be no more complex than needed. The availability of input data to parameterise these models largely drive the degree of complexity that is possible. Keeping models parsimonious can potentially ease interpretation challenges by regulatory bodies. For QIVIVE researchers, the principle of parsimony can also guide research—if a kinetic process is a necessity but cannot be measured, then it may be addressed through either development of a new in vitro method or identification of a set of pre-existing data sufficient to allow inference for new chemicals. Of course, scientific dialogue between regulators and model developers will always be extremely valuable to facilitate the overall understanding and adoption of a modelling approach.

*Default parameters, step 2* “Built-in” values for “default” point estimates of physiological and anatomical parameters may vary amongst platforms/implementations. This highlights the importance to harmonising physiological and anatomical parameters for a specific context of use and an organism. To account for the inter-individual variability and the range of the influential physiological and anatomical parameters, the distribution of those parameters can be defined using the existing data on the biological variability. More importantly, the quality of chemical-specific input parameters is crucial to developing a fit-for-purpose model, especially in the absence of the in vivo data. A guidance document on Good In Vitro Method Practices (GIVIMP) for the development and implementation of in vitro methods for regulatory use in human safety assessment was presented by the OECD (OECD 2018). The guidance aims to reduce the uncertainties in in vitro methods by applying all necessary good scientific, technical and quality practices, documentation, ownership, identity, and applicability domain from in vitro method development to the final in vitro method implementation for regulatory use (OECD 2018). However, more specific guidance documents for the performance of in vitro kinetic studies may be required as well, particularly to guide the selection of appropriate reaction conditions (e.g. linearity checks) (Louisse et al. 2020).

*Good Modelling Practice, step 3* GMP provides as a framework for building fit-for-purpose PBK models (Loizou et al. 2008). GMP should reflect transparency and a stepwise approach on how the model has been setup and how the input parameters are derived. This requires documentation and justifications of the input parameters and modelling steps, and accessibility to the modelling tools. An assessment framework for PBK models was introduced by EFSA 2015 and OECD 2021 (EFSA 2014; OECD Environment 2021), which includes two categories of considerations “Context and Implementation” and “Model Validity”. This may be considered a basis to work with the regulators and the model reviewers towards understanding and adoption of the modelling approach.

*Proof of the predictivity, step 4* WHO (2010) reports that “In PBK modelling, predictions that are, on average, within a factor of 2 of the experimental data have frequently been considered adequate.” (WHO 2010). This threshold for model evaluation was discussed amongst the experts. It was believed that this value is too conservative, since biology accounts (as a minimum) for a factor of 3 (WHO 2005). However, the proposed factor of 2 was related to chemical-specific PBK models. For generic PBK models, a broader applicability domain is required, which makes a stringent limit unrealistic. For example, Punt et al. 2022 revealed that variation in in vivo Cmax values can range between 1.3- and fivefold but can also be as high as 16-fold. Researchers from U.S. EPA have been compiling and analysing in vivo TK data and have shown that replicate in vivo measurements are often (~ 80% of the time) within a factor of two of themselves. These data suggest that a more flexible range in deviation between predicted and observed kinetics needs to be considered (Cook et al. 2022). It is important to remember model simulations and experimental data are subject to uncertainty, which need to be considered to provide a fair comparison between both data (IPCS 2008; Marcus and Robert 1998; WHO 2010). Given that a hard limit for model predictions will be difficult to obtain, more qualitative approaches have been proposed as well, for example, analysing the discrepancies between the model and the data, and determine based on the understanding of the reason for the differences across all observations, whether the model is suitable for extrapolation and, if so, under what conditions.

It is important to realise that when QIVIVE becomes the standard approach for risk assessment, where animal testing would not be allowed anymore, evaluation of the PBK model against in vivo data specific to a given chemical will not be possible (that is, new chemical-specific data will not be collected). PBK models will need to be evaluated against pre-existing in vivo data and the observed performance extrapolated to new chemicals (Cohen Hubal et al. 2019; Wambaugh et al. 2015). Although PBK-based predictions of in vivo blood concentration based on in vitro data have frequently been shown to be sufficiently adequate for a wide range of chemicals, poor model performance is often due to applications outside the original model domain (Punt et al. 2022, 2021a; Wambaugh et al. 2015; Wang 2010; Wetmore et al. 2012). Therefore, it would be important to be able to define a priori if a particular chemical—based on its physicochemical properties and chemical–biological properties—is expected to be within or outside of the PBK model domain (Bell et al. 2018; Wambaugh et al. 2015). In depth analysis of the chemicals that are poorly predicted by PBK models may provide insight into which parameters are of major influence (Wambaugh et al. 2015). So, organisation of existing in vivo data to allow statistical evaluation is a key priority for establishing confidence in QIVIVE (Bell et al. 2018; Sayre et al. 2020; Wambaugh et al. 2018; Watford et al. 2019).

In addition, read-across to structurally similar compounds for which in vivo data for PBK model validation purposes is available would serve as an important way to build confidence (Wambaugh et al. 2015). Read-across approach is proposed as an alternative to in vivo kinetic data to evaluate model predictive performance. The predictive ability on the analogues provides evidence for the applicability of the model to the chemical of interest. So, PBK models for data-poor chemicals may be useful if they are built using a database of structurally diverse data-rich analogues. The rational identification and selection of the analogues have been previously discussed (Ellison 2018; Ellison and Wu 2020; OECD Environment 2021; Paini et al. 2021b). However, this requires more initiatives for data sharing amongst different sectors. Along these lines, the European Partnership for Alternative Approaches to Animal Testing (EPAA) is currently working on assessing the chemical space coverage of existing PBK models (Thompson et al. 2021) and determining methods to identify ‘similar’ chemicals in support of read across.

*Conducting case studies, step 5* Improving regulatory implementation will require case studies. For example, the cosmetics industry conducted several case studies applying PBK modelling in risk assessment (OECD 2020a, b, 2021). These case studies were conducted to demonstrate their applicability in Next Generation Risk Assessment (NGRA) (Berggren et al. 2017), as well as to provide guidance in implementing models into regulatory frameworks. So, additional case studies are required to gain confidence in using PBK modelling for chemical risk assessment.

## Conclusion

With the increasing application of in vitro non-animal NAMs for chemical risk assessment and the concomitant phasing out of animal testing, the use of in vitro assays for quantitative hazard characterisation becomes increasingly important. QIVIVE is a general process translating in vitro concentration–response relationships to in vivo dose–response relationships in humans and animals. PBK models provide a means for QIVIVE and have a substantial role in these extrapolations. Several PBK platforms are available, have a common general approach, and provide similar results, when harmonised inputs are used. Discrepancies in model output are generally due to the choices in model structure and the input data included.

Although bottom-up PBK modelling is increasingly used by the scientific community, regulatory acceptance of model predictions in regulatory risk assessments remains a challenge. Validation of PBK models with in vivo data is still a key step towards regulatory acceptance, yet in vivo PK data for many chemicals are not available. Several steps are recommended and needed to find other means to gain confidence in PBK modelling approaches:

- *Defining the use context*. The regulatory acceptance of bottom-up PBK modelling approaches in risk assessment varies and depends on the risk assessment application and context. For example, their use is better accepted in prioritising risk than establishing reference doses. In human health risk assessment, any lack of data might be addressed if plausible conservative assumptions can be identified. These assumptions should provide a degree of conservatism sufficient to allow for human health protection. Increasing model complexity is unlikely to improve predictive performance and regulatory acceptance, instead fewer mechanisms and fewer parameters often lead to more accurately characterised uncertainties. Scientific dialogues between risk assessment bodies and modellers will help increase the understanding of a model and the value or limitations and facilitate gaining confidence in PBK model simulations, including alignment of terminologies and definitions within the PBK model community.
- *Harmonising physiological input parameters and their distribution* (to assess inter-individual variability). Criteria for good-quality chemical-specific input parameters, especially in the absence of in vivo data, should be defined.
- *Using GMP and a stepwise approach* to develop PBK models to achieve transparency.
- *Developing benchmark datasets and standardised metrics* to evaluate QIVIVE predictions.
- *Carrying out case studies* to gain confidence in PBK model predictions in a regulatory context and serve as critical inputs for developing guidelines.
